# Psychometric assessment of the French European Developmental Coordination Disorder Questionnaire (DCDQ-FE)

**DOI:** 10.1371/journal.pone.0217280

**Published:** 2019-05-23

**Authors:** Sylvie Ray-Kaeser, Evelyne Thommen, Rose Martini, Marianne Jover, Basilie Gurtner, Anne Martine Bertrand

**Affiliations:** 1 Department of Occupational Therapy, School of Social Work & Health Sciences, EESP, HES-SO University of Applied Sciences and Arts Western Switzerland, Lausanne, Switzerland; 2 Department of Social Work, School of Social Work & Health Sciences, EESP, HES-SO University of Applied Sciences and Arts Western Switzerland, Lausanne, Switzerland; 3 Occupational Therapy Program, Faculty of Health Sciences, University of Ottawa, Ontario, Canada; 4 Aix Marseille University, PSYCLE, Aix-en-Provence, France; Hong Kong Polytechnic University, HONG KONG

## Abstract

**Background:**

The Developmental Coordination Disorder Questionnaire’07 (DCDQ’07) is a parent-report measure to identify children at risk for Developmental Coordination Disorder (DCD). We developed a French version of the DCDQ’07 (DCDQ-FE) that has shown excellent inter-language reliability (intraclass correlation coefficient (ICC) = 0.91) and is culturally relevant for use in European countries. The aims of this study were to examine the internal consistency, test-retest reliability, construct validity of the Developmental Coordination Disorder Questionnaire-French European (DCDQ-FE), as well as establish a cut-off score.

**Methods:**

The psychometric properties of the DCDQ-FE were examined with a clinical group of 30 children (mean age: 9.4 years, SD = 2.6) and a control group of 43 children (mean age: 9.1 years, SD = 2.4). Their parents (n = 73) filled out the DCDQ-FE at a first sitting and 70 of them filled it out 38 days later in average for test-retest reliability. The children were assessed using the Movement Assessment Battery for Children-2 (MABC-2) so as to measure the convergent validity of the DCDQ-FE. The cut-off score was determined with an additional sample of 42 children according to scores on the MABC-2 (≥ 16^th^ percentile) (n = 115).

**Results and implications:**

Internal consistency of the DCDQ-FE was excellent (Cronbach’s alpha = 0.96) and test-retest reliability was good (ICC = 0.956) with no differences between scores obtained at the two sittings (p > 0.05). Differences in scores between children in the clinical and control groups (Z = -6.58, p < 0.001) provide evidence of construct validity. The correlation obtained between DCDQ-FE and MABC-2 scores (Spearman’s rho correlation coefficient = 0.802, p < 0.001) supports convergent validity. Using a cut-off of 56, overall sensitivity and specificity were 85.0% and 81.6% respectively (area under the curve = 0.896). The DCDQ-FE is a reliable and valid questionnaire for detecting children who are at risk for DCD in a European-French population of children aged 5 to 15 years old.

## Introduction

Developmental Coordination Disorder (DCD) [1] is a common childhood condition with a prevalence of around 5% to 6% of school-aged children. DCD is idiopathic in nature, occurs across cultures, races and socio-economic conditions, and is more frequent in males than in females. It is a separate disorder, even though it often co-occurs with attention deficit, hyperactivity and learning disorders [2]. The condition is defined by a motor performance that is substantially below expected levels given the child’s chronological age and appropriate type and amount of opportunities for skill acquisition. The motor disturbance significantly interferes with activities of daily living and/or academic achievement [1]. Clinical presentation varies with children experiencing difficulty in performing fine motor activities, such as dressing, eating with use of utensils, drawing, handwriting, and others with gross motor activities such as ball play and sports, which is important for social participation and contact with peers [2, 3, 4].

Numerous studies have highlighted the possible and long-term consequences of this disorder. Children with DCD are at high probability of physical health issues such as reduced fitness and decreased endurance [5, 6], obesity [7], and coronary vascular disease [8]. They choose less diverse and more sedentary and isolated leisure activities, avoiding situations in which they might display their lack of performance [9]. They experience exclusion by peers and limited social participation compared to children without DCD [10]. Their reduced participation becomes more apparent over time and can lead to secondary negative psychosocial and mental health problems, such as low self-esteem, anxiety, depression and behavioural disorders that can affect the whole family system [11]. The vast literature on DCD clearly underlines that this condition affects more than the motor domain and is a burden for society. Since the effects of DCD are so broad, early and accurate identification of the disorder using sound measurement tools is highly recommended [2].

DCD is not easy to identify owing to the heterogeneity of the symptoms, the high co-occurrence of other developmental and behavioural disorders [12, 13], and the large variability in normal motor development and acquisition of activities of daily living [2]. In addition, there is no gold standard measure for DCD as it is not advised to use a single test [14]. There are a few valid tools available to assess the impact of motor difficulties on the performance in everyday activities but identification is much more complicated among a French-speaking population since most of the tools are accessible only in English.

The European Academy for Childhood Disability group (EACD), including the Swiss Society for Developmental Paediatrics, advocates a multidisciplinary approach to the identification of this condition and the use of a multiple assessment procedure [2, 15]. It agrees that the identification of DCD should not be based on just standardized motor tests, which measure body functions and are generally not useful as first-step screening tests since they are expensive and time-consuming. The assessment procedure should include a measure of the interference of the disorder on activities of daily living and participation (e.g. self-care maintenance, school productivity, engagement in leisure and play activities) [2, 16]. The EACD recommends the use of parent or teacher questionnaires as screening tools, in order to document the disturbance of DCD on children’s activities of daily living [2, 15]. Then, if the questionnaire screening has identified a likelihood for DCD, a subsequent evaluation with a standardized norm-referenced motor test needs to be conducted to establish that the motor performance is substantially below expected levels. Because of the high probability of comorbidities in DCD and in order to exclude other medical conditions that may explain motor developmental troubles, it is important that the identification process also address issues of etiology with careful child’s developmental and medical history and examination [2, 15]. The combination of these results with parental reports and motor test results might be the optimal method to determine whether a child meets the DSM diagnostic criteria for DCD.

Several questionnaires have been developed to gather information from parents about the child’s motor performance in everyday activities (Developmental Coordination Disorder Questionnaire’07 (DCDQ’07) [17]; Little DCDQ [18]); teachers (Movement Assessment Battery for Children Checklist (MABC Checklist) [19]); or children themselves (Children Self-perceptions of Adequacy in and Predilection for Physical Activity (CSAPPA) [20]). Among these questionnaires, parental information seems more valid than teacher and child information [2]. The value of parents in detecting developmental concerns and giving valid information about their child’s performance in everyday life has been demonstrated [4, 21, 22].

The Canadian English DCDQ’07 [17] is a revised version of the DCDQ [23] and the best-evaluated parent questionnaire to identify DCD in children between the ages of 5 and 15 [2]. It is a widely used parents’ self-report questionnaire for both research and clinical purposes [24, 25] that is valid, short and access-free. The good psychometric properties of the DCDQ’07 have been confirmed [17]. It has been recommended as supplementary information tool in the diagnosis of DCD [2].

The DCDQ and DCDQ’07 have been cross-culturally adapted in many countries: Australia [26], Brazil [27], China [28], Colombia [29], Japan [30], Germany [31], Italy [32] and The Netherlands [33] among others. Many of these adaptations have shown good internal consistency and excellent test-retest reliability [28, 32]. Their reported sensitivity and specificity values are variable depending on the samples studied and the use of different criteria to define DCD.

Although a Canadian French version of the DCDQ’07, the DCDQ-FC [34] exists, it may not be adapted to the linguistic and cultural specificity of the French-speaking population in Europe. According to Guillemin, Bombardier and Beaton (p. 1420) [35], “an instrument used in a country other than that in which it was developed might require adaptation” owing to “language and cultural differences, and variation in the ways a health condition is expressed”. This is an especially valuable observation for a questionnaire, which aims at understanding the child’s functioning in everyday activities, which are strongly influenced by the culture in which they occur. Moreover, many terms and idioms used in Canada and in French-speaking countries of Europe differ. This difference is observable when reading the questionnaire translated into Canadian French. Such differences are not uncommon in questionnaires and many of them have been adapted both in French European and in French Canadian such as the *Oswestry Disability Index* [36, 37] or the *Disability of Arm*, *Shoulder and Hand* (DASH) questionnaire [38, 39]. Therefore, a cross-cultural adaptation in French European of the DCDQ’07 (DCDQ-FE) and measure of its interlanguage reliability and internal consistency were conducted by Ray-Kaeser and her collaborators [40, 41]. Further psychometric testing of the DCDQ-FE was needed to ascertain that it demonstrates acceptable psychometric properties for identifying children at risk for DCD before being made available for clinical use.

The objectives of the present study were to investigate the internal consistency, test-retest reliability and construct validity of the DCDQ-FE as well as establish a cut-off score using the French version of the MABC-2 as a criterion for diagnosing DCD. A questionnaire with appropriate standardization will help collect information on the DCD-related characteristics necessary to determine if treatment is indicated and to initiate intervention that provides the children with ways to manage their difficulties in order to prevent further consequences of the condition on their health and wellbeing.

## Materials and methods

### Participants

A sample of children and their parents was recruited in the French-speaking area of Switzerland. Typically developing children were recruited in the community (schools, sport centres), through personal contacts and posted information flyers (control group). Children were considered “typically developing” when they were performing within age expectations, with no known motor developmental concerns. The children in the clinical group were recruited from occupational therapy services to which they were referred within the last 6 months due to motor coordination difficulties. To be included, they had to show symptoms of Specific Developmental Disorder of Motor Function (F82) from the International Classification of Diseases of the World Health Organization [42], which is equivalent to DCD. Moreover, the children had to be between 5 years 0 months and 14 years 11 months and attending regular school. Children with any cognitive impairments or neurological disorders (i.e. cerebral palsy, muscular dystrophy) were excluded. To control for possible effect of age or gender in the construct validity assessment, each child in the clinical group was matched with a child in the control group of the same gender and similar age.

To determine a cut-off score, we estimated that a sample of 123 was needed, considering the prevalence of possible DCD in the sample of 40%, sensibility and specificity of 85%, a confidence interval of 95% and a width of precision of 0.10 [43]. Therefore, an additional sample of children and their parents from south of France (French sample) [44] was added to the Swiss sample.

### Measures

The present study included two measures and a demographic questionnaire to collect information on the child’s medical history, development and regular motor activities.

#### French European version of the Developmental Coordination Disorder Questionnaire (DCDQ-07)

The DCDQ-07 asks the parents to rate their child’s motor performance in everyday activities compared with that of typically developing peers through 15 items and usually takes about 10 to 15 minutes to complete. It includes items grouped into three subcategories reflecting areas of motor skills known to present difficulties for children with DCD: 1) control during movement, 2) fine motor / handwriting and 3) general coordination. Each item is scored on a 5-point unipolar scale (from “not at all” to “extremely like your child”). Individual item scores are summed to give a total score, with higher total scores signifying better motor coordination. Cut-off scores according to three age ranges (5.0–7y 11m; 8.0–9y 11m; 10.0 -14y 11m) indicate whether the child is a suspect for DCD or not.

The French European version of the DCDQ’07, the DCDQ-FE, was achieved using guidelines for the cross-cultural adaptation of a self-report measure [45] and was pretested with parents from Switzerland and France [40]. A second study was then conducted to examine the inter-language reliability of the DCDQ-FE with the DCDQ’07 and internal consistency [41]. The inter-language reliability was assessed as good for all three subcategories (intraclass correlation coefficient (ICC) = 0.88–0.89) and the total questionnaire (ICC = 0.91). The internal consistency was high (Cronbach’s alpha (α) = 0.94) and similar to that of the original version. Moreover, the Cronbach’s alpha coefficients, when each item was deleted, indicated that no single item was problematic (α = 0.93–0.94).

#### Movement Assessment Battery for Children second edition (MABC-2)

The MABC-2 [19] is a norm-ranked test designed to measure the child’s gross and fine motor functioning. It is a common tool used for identifying DCD [46]. The test-retest reliability of the instrument is good to excellent and validity is fair to good. It is suitable for children from 3 to 16 years old and evaluates three components: manual dexterity, ball skills, and static and dynamic balance. It takes about 20 to 40 minutes to complete. Raw scores are converted to standard scores for each component. The sum of these standard scores provides a total test score that is then converted to percentile ranks. Children who test above the 16^th^ percentile are considered to have typical motor functioning, between the 16^th^ and the 5^th^ percentile to be at risk of motor difficulties and below the 5^th^ to have motor difficulties. In the present study, the French version of the MABC-2 was used [47].

### Procedure

The parents, mother or father or both, were first asked to complete the DCDQ-FE and the demographic questionnaire. The same parents were invited to complete the DCDQ-FE a second time 30 days later. A reminder letter was sent to ensure the return of all questionnaires.

The children in the control group were tested with the MABC-2 by a research assistant who had received rigorous training in the administration of the test. The children in the clinical group were tested by their occupational therapist. The testing was videotaped for offline scoring according to the test instructions [47] and for checking the congruence of the administration procedure and scoring, in order to limit sources of errors.

### Data analysis

Descriptive statistics were calculated to describe the groups characteristics and the DCDQ-FE scores. Since the items of the DCDQ-FE use an ordinal format response and the sample size was small, and given the non-normal distribution, nonparametric tests were used for comparison. To verify that there was no difference in age between the matched groups (clinical and matched control) a Mann–Whitney U test was performed. To examine if there was a difference in age or gender between the samples from Switzerland and France, Mann–Whitney U and chi-square tests were performed. In addition to examining an effect of age on the DCDQ-FE scores of the children in the clinical and the control groups, Kruskal-Wallis tests were performed with the age ranges that Wilson et al. [17] used to define DCDQ’07 cut-off scores. Finally, Mann–Whitney U tests were performed to examine the effect of gender on the DCDQ-FE scores in each group (clinical and control).

Internal consistency was estimated using Cronbach’s alpha. To evaluate the test-retest reliability of the DCDQ-FE, intraclass correlation coefficients (ICC) (two-way mixed-effect model single measure, absolute agreement type) and their 95% confidence intervals were computed using total and subcategory scores obtained at the first and second completion of the parent questionnaire. Furthermore, the test-retest reliability was estimated for each group separately, since it is recognized that the reliability is specific to the population [48].

To evaluate the construct validity of the DCDQ-FE, the known groups method was used. This consists in comparing scores of a group having the trait and another group that does not [48]. Mann–Whitney U tests were conducted to examine group differences in DCDQ-FE scores. Differences between groups were also examined in each age range. In addition, differential responses (% of scores 4 and 5) between groups were investigated for each item, using a Fisher’s exact test. Finally, to evaluate the convergent validity of the DCDQ-FE, the association between the scores of the questionnaire and those of the MABC-2 was calculated using a Spearman’s rho correlation coefficient (r_s_).

For the above analyses, the statistical significance level was fixed at 0.05.

To establish a cut-off score, a receiver-operating characteristic (ROC) analysis using the MABC-2 16^th^ percentile as the state variable (a score at or below the 16th percentile) was carried out with the Swiss and French samples. Since the sample size was too small to determine a cut-off by age ranges, it was decided to determine a cut-off with the total sample only, according to the best sensitivity and specificity values, and to calculate the positive and negative predictive values with this cut-off by age ranges.

All analyses were conducted using SPSS version 23.

The research project was approved by a research ethics committee (Commission cantonale (VD) d’éthique, 458/2013). Only the child/parent pairs where the child agreed to participate and the parents had signed the consent form were included in the study.

## Results

### Sample characteristics and scores on the DCDQ-FE and MABC-2

The total sample of Swiss participants (n = 73) ranged in age from 5.03 to 14.90 years (mean age = 9.23; SD = 2.45). When matched, the sample (n = 60) also ranged from 5.03 to 14.90 (mean age = 9.38; SD = 2.53). There were no significant age differences (p > 0.05) between groups (clinical vs matched control) (Table 1).

**Table 1:** Swiss groups’ characteristics, DCDQ-FE and MABC-2 scores.

	Clinical group (n = 30)	Matched control group (n = 30)	Control all (n = 43)
Girl/Boy (n)	6/24	6/24	17/26
Age (M (SD), range)	9.42 (2.59), 5.51–14.90	9.33 (2.50), 5.03–14.18	9.10 (2.37), 5.03–14.18
DCDQ-FE total score (M (SD), range)	43.80 (9.92), 24–65	70.63 (4.72), 59–75	70.12 (5.01), 58–75
5.0–7y 11m (n = 10; n = 10; n = 17)	40.40 (11.09), 24–62	69.80 (4.64), 62–75	69.53 (4.98), 58–75
8.0–9y 11m (n = 9; n = 9; n = 11)	42.89 (9.58), 27–54	72.44 (4.25), 65–75	71.82 (4.85), 63–75
10.0–14y 11m (n = 11; n = 11; n = 15)	47.64 (8.52), 37–65	69.91 (5.15), 59–75	69.53 (5.50), 59–75
Girls (n = 6; n = 6; n = 17)	45.00 (7.32), 36–53	70.83 (4.36), 65–75	69.59 (5.45), 58–75
Boys (n = 24; n = 24; n = 26)	43.50 (10.58), 24–65	70.58 (4.89), 59–75	70.46 (4.74), 59–75
MABC-2 total percentile (median (lower-upper quartile), range)	2 (1–13), 0.10–50	69 (50–93), 9–99	63 (50–84), 9–99

Among the 30 children in the clinical sample, 10 children were born preterm (mean of 35 gestational weeks). Seven children showed behavioral issues, ten had peer relationship problems and 13 had signs of hyperactivity, according to their parents.

The additional sample of participants from France used to determine a cut-off score (n = 42) ranged in age from 5.70 to 14.90 years (mean age = 8.98; SD = 2.02) and comprised 13 girls and 29 boys. Among them, 22 children were referred for motor developmental difficulties. There were no significant differences in age or gender from the Swiss sample (p > 0.05).

Table 1 also presents the DCDQ-FE and MABC-2 scores of the Swiss sample. There were no differences in DCDQ-FE scores between age ranges in the clinical group (p = 0.184) or in the control groups (matched: p = 0.269; control all: p = 0.179). Furthermore, there were no differences in DCDQ-FE scores between gender in the clinical group (p = 0.781) as well as in the control groups (matched: p = 0.900; control all: p = 0.616).

### Internal consistency and test-retest reliability

Internal consistency was excellent with a Cronbach’s alpha of 0.96 for the total score (n = 73). All items were moderately to highly correlated with the total score (0.673–0.836) and Cronbach’s alpha coefficient for the total score remained excellent if items were deleted (Table 2).

**Table 2:** Results of the internal consistency analysis and differences in percentage of scores 4 and 5 between the clinical and the control group.

	Internal consistency		Difference in the percentage of scores of 4 or 5 between groups		
	r item-total (n = 73)	α if item deleted (n = 73)	% Clinical group (n = 30)	% Matched control group (n = 30)	Chi-square
1. Throws ball	0.822	0.957	0.30	0.90	22.50
2. Catches ball	0.824	0.957	0.23	0.90	27.15
3. Hits ball/birdie	0.760	0.958	0.13	0.80	26.79
4. Jumps over	0.836	0.957	0.47	0.97	18.47
5. Runs	0.741	0.959	0.50	1.00	20.00
6. Plans activity	0.673	0.960	0.63	1.00	13.47
7. Writing fast	0.790	0.958	0.30	0.97	28.71
8. Writing legibly	0.732	0.959	0.43	1.00	23.72
9. Effort and pressure	0.823	0.957	0.27	0.93	27.78
10. Cuts	0.768	0.958	0.27	0.97	31.09
11. Likes sports	0.730	0.959	0.50	0.90	11.43
12. Learning new skills	0.818	0.957	0.30	0.97	28.71
13. Quick and competent	0.813	0.957	0.27	1.00	34.74
14. Skillful	0.758	0.958	0.20	1.00	40.00
15. Sits upright	0.747	0.959	0.20	0.93	32.85

All differences in percentage of scores 4 and 5 between the clinical and the control group were significant (p < 0.002).

While a 30-day interval was planned to estimate the test-retest reliability, mean test-retest period was 38 days (SD = 17). All parents of the Swiss sample completed the DCDQ-FE twice except two in the clinical group and one in the control group. The intraclass coefficient was 0.865 for the clinical group (n = 28), 0.814 for the control group (n = 42) and 0.956 for the total sample (n = 70), with no differences between scores at the two different points measurement (p > 0.05), indicating a good reliability. The ICC and their 95% confidence intervals are shown in Table 3.

**Table 3:** Test-retest subcategories and total scores reliability.

	ICC_(2.1)_ (95% CI)		
	Clinical group (n = 28)	Control all (n = 42)	Total sample (n = 70)
Control during movement	0.825 (0.656–0.915)	0.787 (0.636–0.880)	0.913 (0.864–0.945)
Fine motor/Handwriting	0.749 (0.530–0.875)	0.765 (0.604–0.866)	0.916 (0.868–0.947)
General coordination	0.885 (0.768–0.945)	0.787 (0.639–0.879)	0.955 (0.929–0.972)
Total	0.856 (0.713–0.931)	0.814 (0.681–0.895)	0.956 (0.930–0.972)

### Construct validity

Construct validity was addressed by comparing the DCDQ-FE total scores of the clinical group (n = 30) with scores from the matched control children (n = 30). Scores of the children in the control group were higher, showing a better performance than the children in the clinical group (Z = -6.58, p < 0.001). Similar results were found when the analysis was performed separately for each age range (1: Z = -3,75, p < 0.001; 2: Z = -3,61, p < 0.001; 3: Z = -3,85, p < 0.001). The difference in the percentage of scores of 4 or 5 between groups was significant for each item (Table 2).

Construct validity was also addressed by examining the relation between the DCDQ-FE total scores with MABC-2 total percentile scores of the children in the clinical and matched control groups (n = 60). DCDQ-FE total scores were significantly correlated to the MABC-2 total percentile scores (r_s_ = 0.802, p < 0.001).

### Cut-off score

The optimal cut-off score for the DCDQ-FE was 56 (area under the curve (AUC) = 0.896 (CI_95_ = 0.841–0.951)), with a sensitivity of 0.85 and specificity of 0.81, regardless of the age range of the children in the Swiss sample combined with the French sample (n = 115). The positive and negative predictive values were 0.71 and 0.91, respectively. The sensitivity and specificity values as well the positive and negative predictive values for each age range using a cut-off at 56 are presented in Table 4.

**Table 4:** Sensitivity, specificity of the DCDQ-FE using a cut-off at 56.

	Sensitivity	Specificity	Predictive positive value	Predictive negative value
5.0–7y 11m	0.75	0.91	0.86	0.83
8.0–9y 11m	1.00	0.74	0.63	1.00
10.0–14y 11m	0.83	0.81	0.67	0.92

Table 5 shows how the DCDQ-FE classified children as suspect or probably not DCD compared to the MABC-2 (≤ 16^th^ percentile) using the DCDQ-FE cut-off score (56) and the DCDQ’07 cut-off score (53) regardless of age for the Swiss and French samples (n = 115). The agreement between the DCDQ-FE and DCDQ’07 cut-off scores for the whole sample was 94.8%.

**Table 5:** Classification of DCD with the MABC-2 (< = 16^th^ percentile) using the DCDQ-FE and DCDQ’07 cut-off scores (n = 115).

		MABC-2	
		Suspect DCD (≤ 16^th^ percentile)	Probably not DCD (> 16^th^ percentile)
DCDQ-FE	Suspect DCD (≤56)	34^a^	14^b^
	Probably not DCD (>56)	6^c^	61^d^
DCDQ-07	Suspect DCD (≤53)	30^a^	12^b^
	Probably not DCD (>53)	10^c^	63^d^

^a^ true positive;

^b^ false positive;

^c^ false negative;

^d^ true negative

With a cut-off at 56, the sensitivity (0.85) and specificity (0.81) values of the DCDQ-FE reached the recommended standard of the American Psychological Association (APA) for sensitivity (0.80) although not for specificity (0.90), and were higher than those of the DCDQ’07, respectively 0.84 and 0.70 [17].

## Discussion

The objectives of the present study were to investigate the internal consistency, test-retest reliability and construct validity of the French European version of the DCDQ’07, the DCDQ-FE, and establish a cut-off score for identifying children with DCD. Previous studies have demonstrated that the DCDQ-FE is culturally adapted for the French European language and culture [40], and the good interlanguage reliability and internal consistency of the tool [41].

The present study showed that the DCDQ-FE has sound psychometric values. The results for internal consistency showed excellent homogeneity and confirmed that all items measure motor coordination. Coefficient alpha (0.96) was comparable to that of the original version [17] and our preliminary study [41] with 30 children.

The values of the intraclass coefficient correlation indicated that the test-retest reliability is excellent for the overall questionnaire and total sample, and is able to measure with stability parental perceptions of their child’s motor performance over time. The reliability is good considering the total for each group. Regarding the three subcategories separately within each group, the reliability is moderate. This could be explained by the small sample sizes.

The DCDQ-FE total scores showed significant differences between groups, with the children in the clinical group presenting poorer motor performance than those in the control group, as would be expected. Moreover, all items significantly distinguished the children in the two groups when the percentages of time scores 4 and 5 assigned to all items were compared.

The high association between the DCDQ-FE and the MABC-2 scores indicates that the DCDQ-FE addresses motor performances that are specifically difficult for children at risk of or having definite motor difficulties. The questionnaire can be used to decide whether a child needs further motor assessment with the MABC-2. As recommended, a child that is categorized “suspect for DCD” on the DCDQ-FE score should then be assessed with a norm-referenced test to confirm the motor difficulties [2, 15].

There was no significant difference in the DCDQ-FE mean scores between children in the three age ranges, although Wilson et al. [17] found a correlation between total score and age. Even if children are likely to become more performing in motor abilities with age, such correlation was not expected given that the DCDQ-FE asks the parents to compare their child’s abilities to those of their peers. Moreover, our findings did not support a main effect of gender on the DCDQ-FE scores as in the studies of Nakai et al. [30] and Tseng et al. [28]. This might be best explained by a cultural difference. Indeed, items of the DCDQ’07 were developed to measure motor skills with minimal bias from gender [17]. Very few changes needed to be made to the DCDQ-FE content since the activities (except for the use of a bat in item 3) are also experienced by boys and girls in Western Europe.

Although the specificity values of the DCDQ-FE did not reach the APA standards of 0.90 for children aged 8 to 15 years, and since the questionnaire is meant to identify children at risk for DCD in a clinical population, it is more appropriate to have higher sensitivity, or the capacity to identify all those who are at risk for DCD than specificity, or the capacity to identify children without the condition [49]. With the most appropriate overall cut-off score at 56, the sensitivity of the DCDQ-FE without adjusting the scores for ages is 85%, which is higher than that of the original DCDQ’07 (81%).

The sensitivity of the DCDQ’FE for children aged 5.0 to 7.11 years was moderate (75%) and similar to the DCDQ’07 [17]. Therefore, confirmatory testing is specifically necessary for this age range. However, specificity was high (91%), which implies that few false positives, or children incorrectly identified as DCD suspects, can occur.

The overall cut-off score of 56 was higher than the DCDQ’07 (53). This might be explained because the number scale 1 to 5 was found problematic when the DCDQ-FE was pretested [40]. Feedback from parents indicated that the number 3 was mainly used to say “my child performs like the others”. To rectify this, further explanation on the meaning of the scale (score 5 meaning “my child performs like the others”) was provided in the introduction of the questionnaire, which might explain why overall scores are higher with this version.

Further investigation of the predictive validity showed that six children in the Swiss-French sample obtained high scores on the DCDQ-FE (not suspect) and were found to be under the 16^th^ percentile on the MABC-2 (false negative). It should be noted that three of these children were 5.0 to 7.11 years old, an age when there is still a great deal of variation in the motor coordination development. Another possible explanation is that these children may have lacked motivation and concentration when tested with the MABC-2. Moreover, it might also be possible that they had motor difficulties that did not impact their daily motor performance significantly or that their parents did not notice such difficulties.

The DCDQ-FE appears to provide a quick first-step screening questionnaire, easy to administer. It is the first valid parental questionnaire in French developed for identifying children at risk of DCD among a European-French population. It can be used to support a diagnosis of DCD by evaluating criterion II of the EACD guidelines [2]. It is recommended to be used by health professionals "in a clinical setting as supplementary information in the diagnosis of children with DCD” [2, p. 19]. It can assist them in referring the children at risk of DCD for further testing as well as in documenting the impact of the condition on children’s everyday activities. It will be available for download at http://www.dcdq.ca.

The Little DCDQ questionnaire [18], based on the DCDQ’07 for screening preschool children, has also been adapted into European French. With both questionnaires, it will be possible to ensure intervention as soon as possible to prevent motor delays, lessen problems in everyday activities and avoid secondary emotional and social consequences.

One major limitation of the present study is the small number of participants, which might compromise the generalizability of the results and did not allow a confirmatory factor analysis to be conducted. Moreover, only one measure (MABC-2) was used to identify children at risk of DCD. This may have influenced the sensitivity and predictivity values and limited the determination of cut-off scores.

Further research on the DCDQ-FE psychometric properties should focus on the sensitivity and specificity for the younger age range using a larger clinical sample of children who meet the diagnostic criteria and are representative of children in French-speaking European countries. It would be worthwhile to control for comorbidities as they exacerbate the risk of DCD. Finally, a longitudinal study using the DCDQ-FE could be warranted to track a child’s motor performance over time.

## Conclusions

Early identification is critical to implement adequate and timely intervention so as to prevent the consequences of the disorder since its burden is considerable both for children and for society. There are few simple and quick measures in French currently available to identify children at risk or with motor impairment, such as DCD. This study addressed aspects of reliability and validity of the French European version of the DCDQ’07, the DCDQ-FE. The results showed that the DCDQ-FE is highly correlated with the French version of the MABC-2 and can discriminate children with and without motor impairment. The DCDQ-FE is a psychometrically sound instrument that shows promise for helping health professionals to identify children eligible for further testing and interventions.

## Supporting information

S1 Data DCDQ-FE and MABC-2 scores. (XLSX)
